# Concurrent Cerebellar and Spinal Infarction in Congenital Hypofibrinogenemia: A Report of a Rare Case

**DOI:** 10.7759/cureus.90881

**Published:** 2025-08-24

**Authors:** Mustafa Al Hassani, Zaid Al Hassani, Walaaeldin ElKhalifa, Hina Abbasi, Adel Elbery

**Affiliations:** 1 Internal Medicine, Tawam Hospital, Al Ain, ARE; 2 College of Medicine, University of Sharjah, Sharjah, ARE; 3 Internal Medicine, Sheikh Tahnoon Medical City, Al Ain, ARE; 4 Neurology, Sheikh Tahnoon Medical City, Al Ain, ARE; 5 Radiologist, Sheikh Tahnoon Medical City, Al Ain, ARE

**Keywords:** cerebellar stroke, fibrinogen replacement, hypofibrinogenemia, spinal stroke, thrombosis

## Abstract

Congenital hypofibrinogenemia is a rare autosomal recessive disorder characterized by significantly reduced plasma fibrinogen levels and a predisposition to bleeding. Paradoxically, affected individuals also face an increased risk of thrombotic events. However, simultaneous involvement of both the cerebellum and spinal cord due to ischemic strokes is exceedingly rare. We report the case of a 41-year-old woman with a known history of congenital hypofibrinogenemia and prior cerebral hemorrhage, who presented with sudden-onset numbness in all four limbs, rapidly progressing to tetraplegia and sensory loss at the C4 level. Episodes of vertigo and chronic cervical pain preceded these symptoms. Coagulation studies revealed a critically low fibrinogen level (<0.30 g/L), markedly elevated international normalized ratio (INR >10.00), and prolonged activated partial thromboplastin time (APTT >180 seconds). Neuroimaging confirmed a subacute infarction in the right cerebellar hemisphere and ischemic changes in the cervical spinal cord from C2 to C5. Management included fibrinogen replacement therapy, high-dose corticosteroids, and non-invasive ventilatory support; anticoagulation was withheld due to the substantial hemorrhagic risk. This case exemplifies the clinical complexity of managing patients with hypofibrinogenemia, where severe coagulation deficits may paradoxically lead to ischemic events. To our knowledge, this is the first reported case of concurrent cerebellar and spinal cord infarctions in a patient with congenital hypofibrinogenemia, underscoring the need for heightened clinical vigilance and the development of evidence-based treatment protocols.

## Introduction

Fibrinogen is a key glycoprotein synthesized by the liver and essential for hemostasis. It supports platelet aggregation and serves as the precursor to fibrin, which forms the structural framework of stable blood clots during secondary hemostasis [1]. Inherited fibrinogen disorders, including hypofibrinogenemia (a quantitative deficiency) and dysfibrinogenemia (a qualitative abnormality), are rare autosomal recessive conditions, with an estimated prevalence of one to two cases per million individuals worldwide [1,2].

Although hypofibrinogenemia is typically associated with bleeding due to inadequate fibrin clot formation at plasma fibrinogen levels below 1.5 g/L, recent evidence highlights an unexpected tendency toward thrombotic events. Arterial and venous thromboembolism have been reported in up to 20% of affected patients [3,4]. This paradox arises from genetic mutations in *FGA*, *FGB*, or *FGG*, which impair the structure and function of fibrinogen. These mutations can reduce thrombin binding, interfere with normal fibrinolytic activity, and enhance platelet aggregation, collectively contributing to a prothrombotic environment despite overall hypocoagulability [3,5].

Ischemic stroke in the context of hypofibrinogenemia remains poorly characterized, with fewer than 15 cases reported in the literature [6]. Proposed mechanisms include accumulation of unbound thrombin, impaired tissue plasminogen activator (tPA)-mediated fibrinolysis, and altered platelet-fibrinogen interactions that increase clot stability [3,7]. To date, concurrent infarction of both the cerebellum and spinal cord has not been documented in patients with this condition. Such presentations pose significant diagnostic and therapeutic challenges, particularly given the lack of evidence-based treatment guidelines. Clinical management must account for the dual risks of bleeding and thrombosis. Anticoagulation may increase the likelihood of hemorrhage, while fibrinogen replacement alone may not adequately address the underlying thrombotic risk [8].

## Case presentation

A 41-year-old woman with a known diagnosis of congenital hypofibrinogenemia, followed regularly at a tertiary hematology clinic, presented to the emergency department with a sudden onset of bilateral numbness and weakness in both upper and lower limbs. These symptoms developed approximately 1.5 hours prior to arrival. Over the preceding 10 days, she had experienced worsening chronic neck pain, which prompted outpatient evaluation and cervical spine radiography. She had also reported an episode of transient vertigo, dizziness, and imbalance 10 days before admission, which resolved spontaneously and was not investigated further at the time.

The patient was diagnosed with congenital hypofibrinogenemia during childhood at a different tertiary center. Her condition was confirmed through hematological assessment and long-term follow-up. She comes from a consanguineous family, as her parents are first cousins, and out of nine siblings, three have been diagnosed with the same condition. This pattern is consistent with an autosomal recessive mode of inheritance.

Within 12 hours of admission, the patient developed acute sensory loss in all 4 limbs and progressive bilateral lower limb weakness. This rapidly advanced over the following two hours to complete tetraplegia, with a sensory level at the C4 dermatome. Additional symptoms included a mild headache and hypophonia. Cognition and cranial nerve function remained intact, and she denied any recent trauma, urinary retention, or bowel incontinence.

On neurological examination, the patient was alert and oriented, with a Glasgow Coma Scale score of 15. Cranial nerves were intact, muscle tone was increased in all extremities, and there was complete sensory loss below the C4 level.

Laboratory investigations revealed profound coagulopathy. Her international normalized ratio (INR) was greater than 10.00, activated partial thromboplastin time (APTT) exceeded 180 seconds, and her fibrinogen level was critically low, measuring less than 0.30 g/L (Table 1). Venous blood gas analysis showed respiratory acidosis with hypercapnia (pCO₂: 51.3 mmHg) and hypoxemia (pO₂: 64.9 mmHg, oxygen saturation: 90.6%) (Table 2).

**Table 1:** Coagulation profile demonstrating critical hypofibrinogenemia and coagulopathy PT: prothrombin time; INR: international normalized ratio; APTT: activated partial thromboplastin time

Coagulation Profile (Components)	Value	Normal Range
PT	120 s	10 – 14 s
INR	>10	0.8 – 1.2
APTT	180 s	25 – 40 s
Fibrinogen	< 0.30 g/L	2 – 4 g/L

**Table 2:** Venous blood gas (VBG) analysis revealing respiratory acidosis and hypoxemia

Blood Gas Analysis (Components)	Value	Normal Range
pH	7.33	7.35 – 7.45
pCO₂ (arterial)	51.3 mmHg	35 – 45 mmHg
pO₂ (arterial)	64.9 mmHg	75 – 100 mmHg
HCO₃⁻	27 mmol/L	22 – 26 mmol/L
O₂ Saturation (SaO₂)	90.6%	95 – 100 %

**Figure 1:**
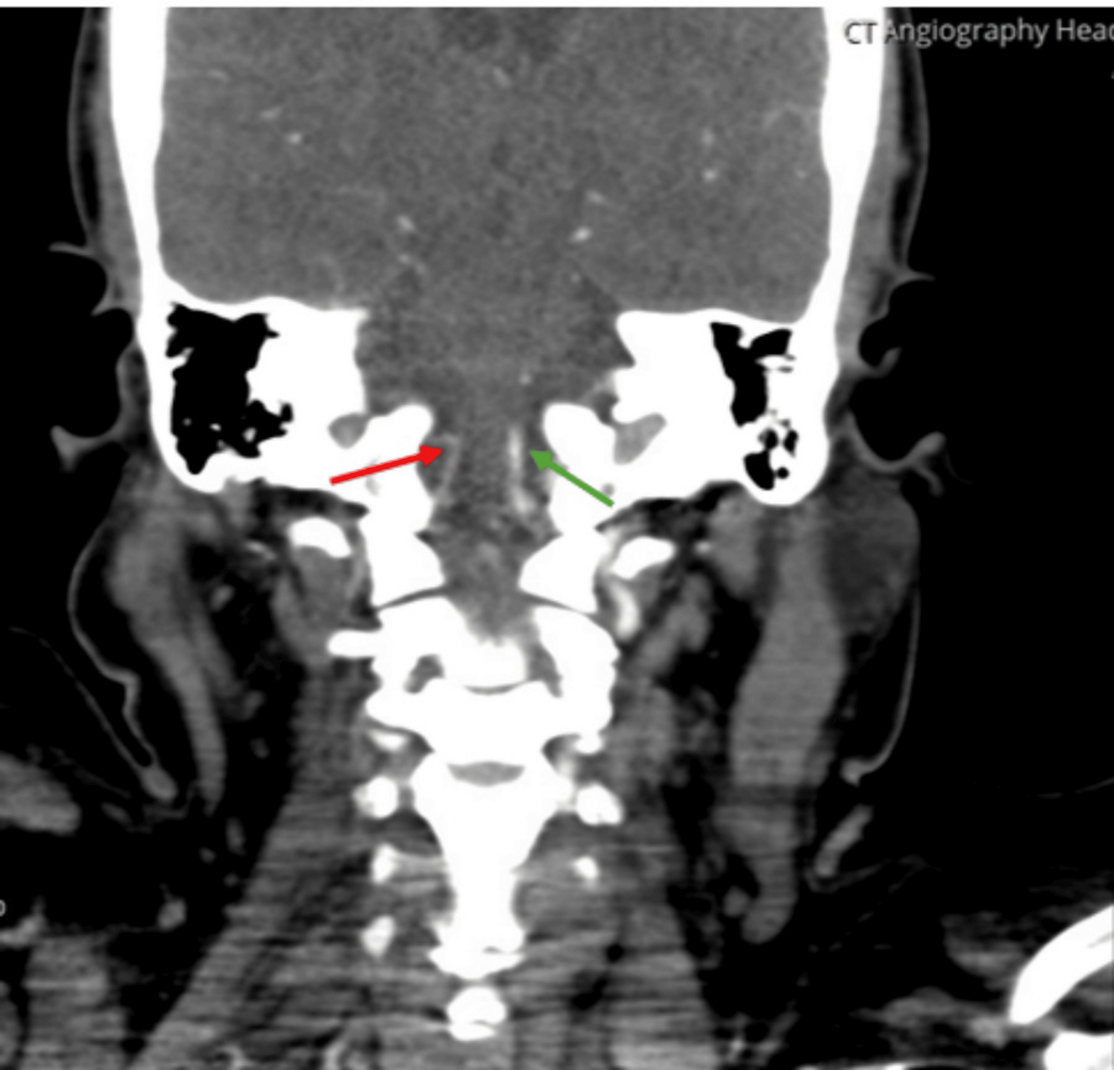
Right vertebral artery dissection on CTA Computed tomography angiography (CTA) of the neck shows reduced enhancement and narrowing of the right vertebral artery (red arrow) compared to the normal left vertebral artery (green arrow), consistent with acute dissection.

**Figure 2:**
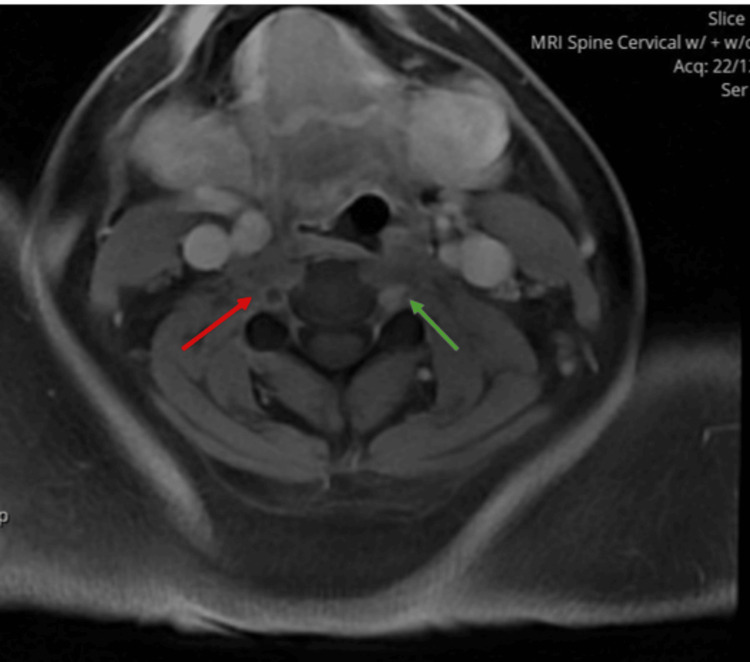
Filling defect in the right vertebral artery on MRI Magnetic resonance imaging (MRI) of the neck shows a filling defect within the right vertebral artery (red arrow), suggestive of thrombus formation or progression of dissection, in contrast to the normal enhancement of the left vertebral artery (green arrow).

Computed tomography angiography (CTA) of the neck, performed on day 1, showed reduced enhancement and attenuation of the right vertebral artery compared to the left, consistent with acute vertebral artery dissection (Figure 1). Follow-up magnetic resonance imaging (MRI) of the neck on day 3 demonstrated a filling defect in the right vertebral artery, suggestive of thrombus formation or progression of the dissection (Figure 2). Brain MRI revealed a subacute infarct in the right cerebellar hemisphere, while cervical spine MRI on day 2 showed a longitudinal hyperintense signal in the posterior spinal cord extending from C2 to C5, consistent with early ischemia. A repeat MRI on day 3 showed increased signal intensity in the same region, confirming progression to spinal cord infarction.

**Figure 3:**
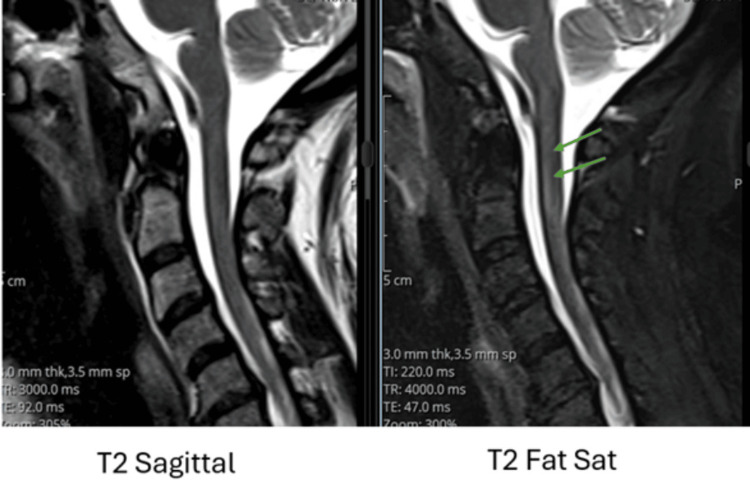
Initial spinal cord hyperintensity on magnetic resonance imaging (MRI) of the cervical spine Sagittal T2-weighted and T2 fat-saturated MRI sequences of the cervical spine show a longitudinal linear hyperintense signal in the posterior spinal cord from C2 to C5 (green arrows), suggestive of early ischemic or inflammatory changes.

**Figure 4:**
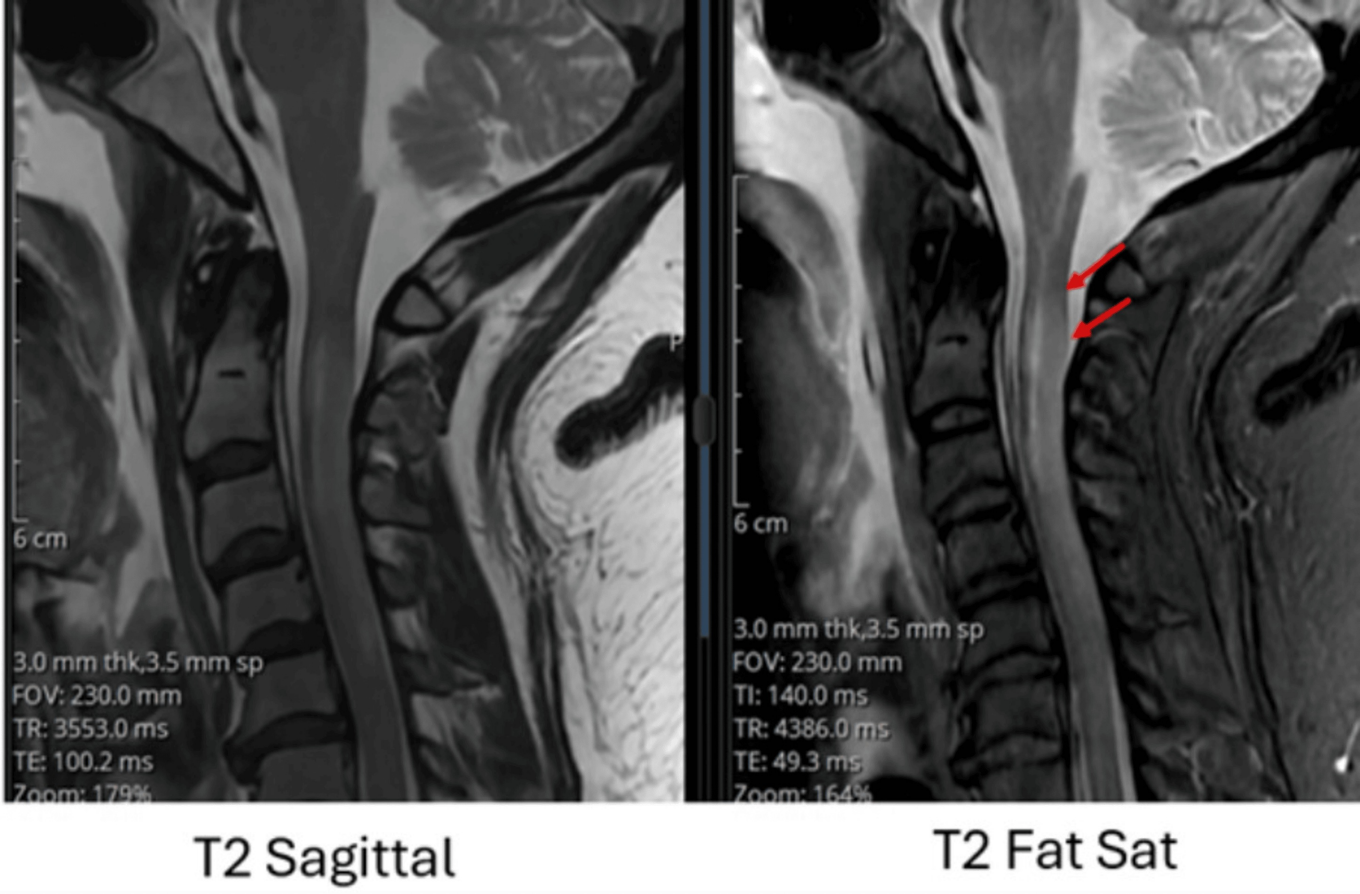
Progression of spinal cord hyperintensity on follow-up magnetic resonance imaging (MRI) of the cervical spine Follow-up sagittal T2-weighted and T2 fat-saturated MRI sequences of the cervical spine show increased conspicuity of the longitudinal hyperintense signal in the posterior spinal cord from C2 to C5 (red arrows), consistent with evolving spinal cord ischemia.

On day 2, cervical spine MRI using sagittal T2-weighted and T2 fat-saturated sequences revealed a longitudinal hyperintense signal in the posterior spinal cord extending from C2 to C5, suggestive of early ischemic or inflammatory changes (Figure 3). A follow-up MRI performed on day 3 demonstrated increased conspicuity and extension of the same hyperintense signal in this region, consistent with progression to spinal cord infarction (Figure 4). This interval change illustrates the utility of serial MRI in confirming evolving spinal cord ischemia and guiding early therapeutic decision-making.

**Figure 5:**
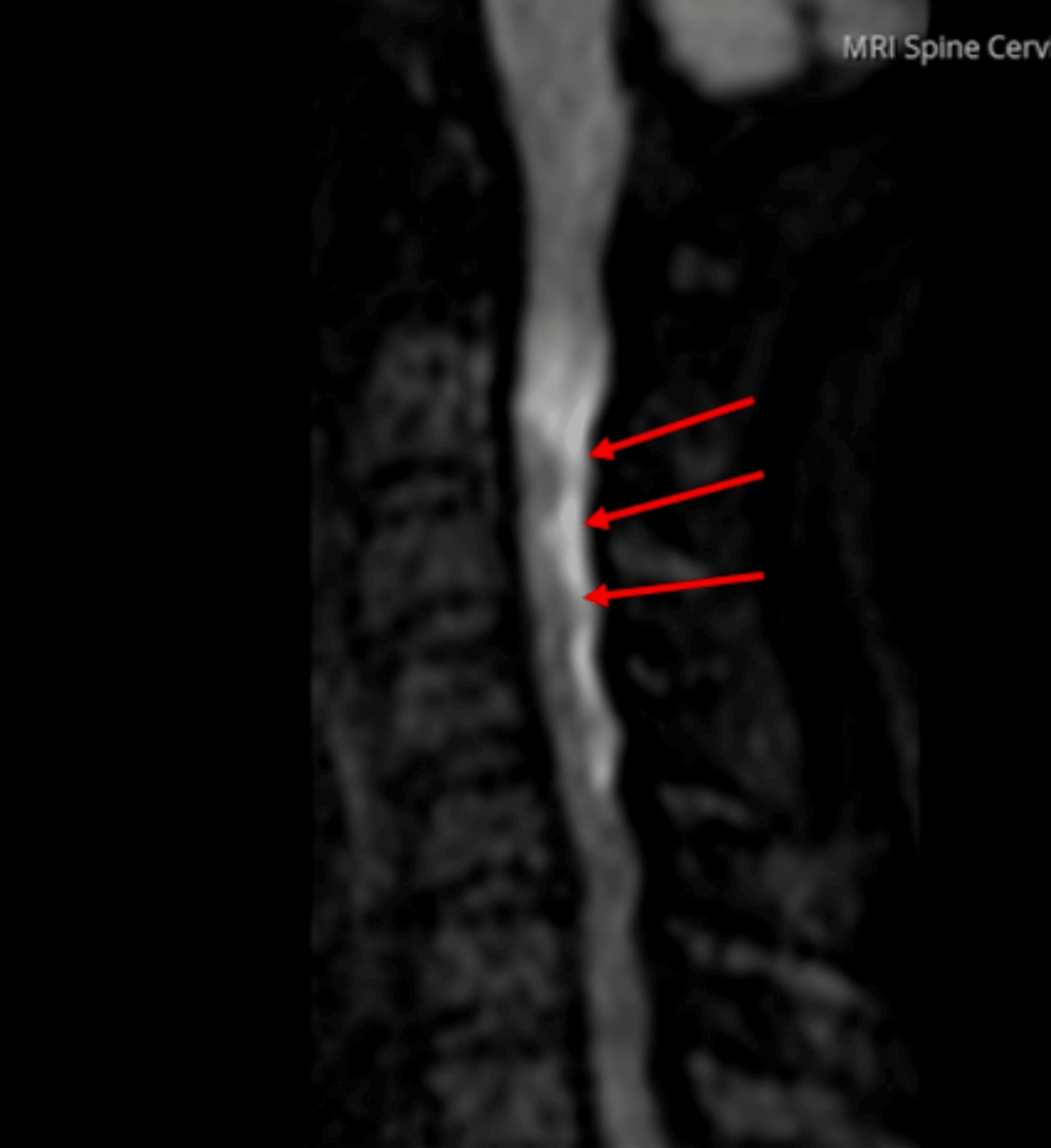
Spinal cord infarction on magnetic resonance diffusion-weighted imaging (MRI-DWI) of the cervical spine DWI of the cervical spine shows a longitudinal hyperintense signal with restricted diffusion in the posterior spinal cord extending from C2 to C5 (red arrows), consistent with acute spinal cord infarction.

**Figure 6:**
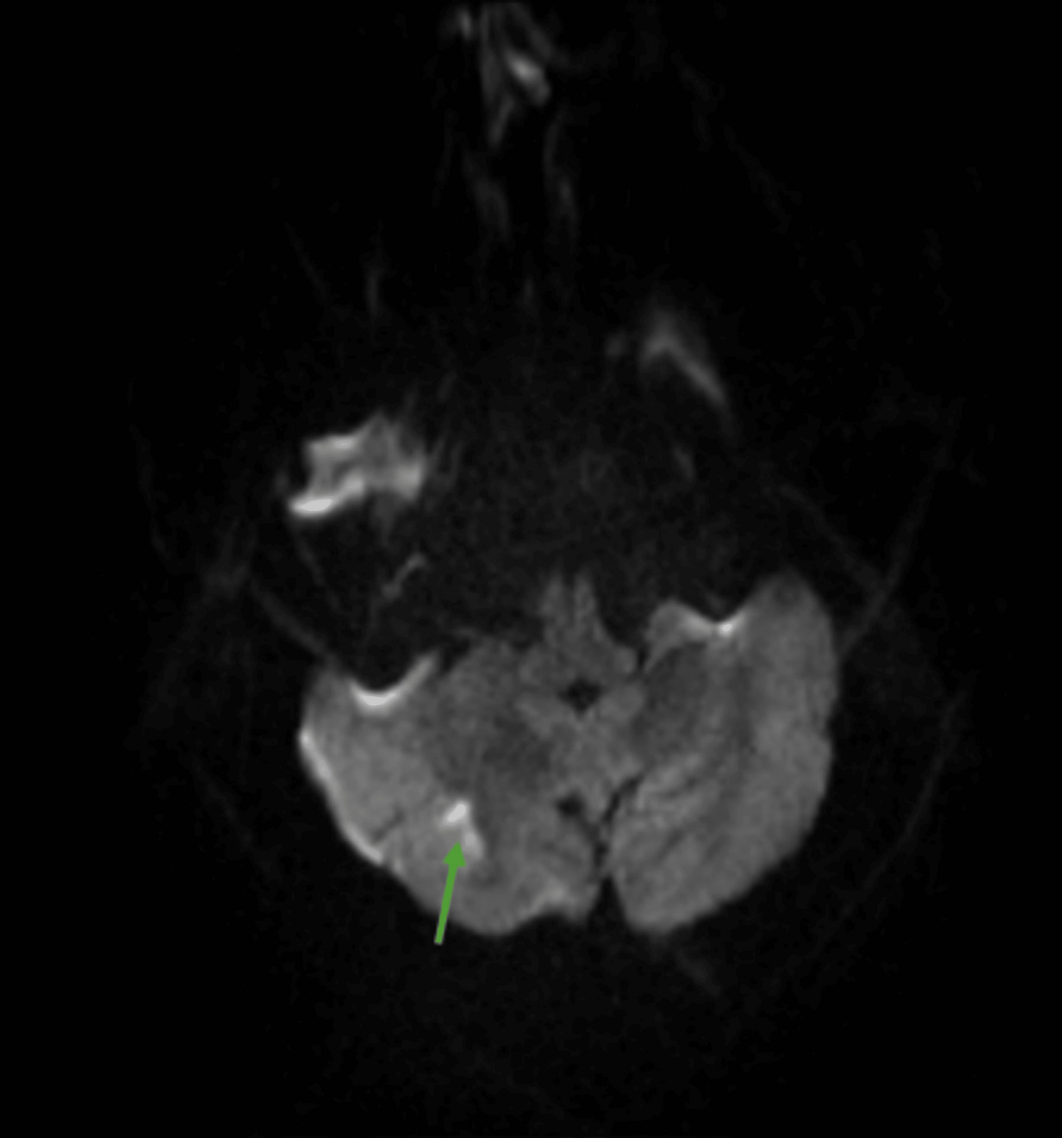
Cerebral infarction on magnetic resonance diffusion-weighted (MRI-DWI) imaging of the brain Diffusion-weighted imaging (DWI) of the brain shows a small focal area of restricted diffusion in the right cerebral hemisphere (green arrow), consistent with an acute ischemic infarct.

On day 3, diffusion-weighted imaging (DWI) of the cervical spine revealed a longitudinal hyperintense signal with restricted diffusion in the posterior spinal cord extending from C2 to C5, confirming the diagnosis of acute spinal cord infarction (Figure 5). In addition, a brain MRI performed on day 2 demonstrated a small area of restricted diffusion in the right cerebellar hemisphere, indicative of a concurrent acute cerebral infarct (Figure 6). Together, these findings confirmed multifocal ischemic events, with spinal cord involvement being the predominant driver of the patient's clinical presentation.

Guillain-Barré syndrome (GBS) was excluded due to the absence of clinical or imaging features typical of demyelinating polyneuropathy. Lumbar puncture (LP) was deferred due to elevated bleeding risk from critical hypofibrinogenemia and increased INR levels.

Immediate management included bi-level positive airway pressure (BiPAP) to address hypercapnia, followed by fibrinogen replacement therapy with 4000 milligrams (mg) of fibrinogen concentrate and four units of fresh frozen plasma (FFP) prior to invasive procedures. Pulse steroid therapy (intravenous methylprednisolone 1 gram (g) per day for 5 days with gradual tapering) was initiated to reduce spinal cord edema. Anticoagulation for thromboprophylaxis was contraindicated due to the bleeding risk.

Her modified Rankin Scale (mRS) score on admission was 5, indicating severe disability with complete dependence for all activities of daily living. The patient required tracheostomy and mechanical ventilation via a life support ventilator (LTV) for acute respiratory failure, necessitating transfer to a long-term care facility for ongoing rehabilitation, including physiotherapy and respiratory support.

## Discussion

Inherited fibrinogen disorders, such as hypofibrinogenemia, are clinically heterogeneous and may present with a range of manifestations, from asymptomatic laboratory abnormalities to life-threatening hemorrhages or thromboses [1]. The case presented here exemplifies the paradoxical risk of thrombosis in a patient with severe congenital hypofibrinogenemia, evidenced by the development of concurrent cerebellar and spinal cord infarctions despite critically low fibrinogen levels, markedly elevated INR, and prolonged APTT.

The prothrombotic phenotype observed in some patients with fibrinogen deficiency is thought to result from multiple interacting mechanisms. Mutations in the *FGA, FGB, *and *FGG *genes, which affect the structure and synthesis of fibrinogen, can impair thrombin binding and delay fibrin formation [3]. In the absence of adequate fibrin scaffolding, thrombin remains in circulation at elevated levels, promoting excessive platelet activation and aggregation [4]. Additionally, abnormal fibrin architecture impairs the binding and activity of tissue plasminogen activator (tPA), limiting fibrinolysis and thereby increasing the persistence of thrombi [5].

The diagnosis of thrombotic events in hypofibrinogenemia can be delayed or confounded by the co-existence of hemorrhagic risk and nonspecific symptoms. Conventional coagulation assays, such as prothrombin time (PT), APTT, and fibrinogen levels, may confirm the diagnosis of coagulopathy but provide limited insight into thrombotic risk or clot quality [6]. More advanced techniques, including thromboelastography, scanning electron microscopy of clot ultrastructure, and genetic analysis of fibrinogen subunits, may offer greater insight into clot stability and fibrinolytic resistance [7]. In this case, MRI of both the brain and cervical spine was essential for confirming multifocal infarctions, and serial imaging provided critical evidence of infarct progression. Lumbar puncture was appropriately deferred due to coagulopathy and high bleeding risk.

Management of thrombosis in hypofibrinogenemia remains challenging due to the competing risks of hemorrhage and ischemia. Fibrinogen replacement is generally indicated to maintain hemostasis, particularly before invasive procedures or in the setting of active bleeding. However, replacement therapy may also exacerbate thrombotic risk by restoring fibrin formation in a procoagulant environment [8]. In this case, the patient received fibrinogen concentrate and fresh frozen plasma to correct her coagulopathy, along with high-dose corticosteroids to reduce spinal cord edema. Anticoagulation was avoided due to the patient’s history of intracranial hemorrhage and extreme coagulation derangement. This reflects a common clinical dilemma in the management of such patients, in which therapeutic strategies must be individualized and guided by a multidisciplinary team.

While low-molecular-weight heparin is sometimes used in thrombotic phenotypes of fibrinogen disorders, its use remains controversial, particularly in the absence of robust monitoring parameters [9]. Future therapeutic directions may include targeted thrombin inhibitors or engineered fibrinogen molecules with preserved clot-forming function and improved fibrinolytic regulation. Until such therapies are validated, careful balancing of bleeding and thrombotic risks remains critical.

This case adds to the limited literature on thrombotic complications in hypofibrinogenemia and is, to our knowledge, the first to report simultaneous cerebellar and spinal cord infarction in this setting. It underscores the importance of early neuroimaging in patients with atypical neurologic presentations and known bleeding disorders. Furthermore, it highlights the urgent need for consensus guidelines that address the dual hemostatic challenges in these rare but high-risk patients.

## Conclusions

This case illustrates the clinical complexity of managing thrombotic events in patients with congenital hypofibrinogenemia. Although the disorder is primarily associated with bleeding, this patient developed simultaneous cerebellar and spinal cord infarctions despite having severely reduced fibrinogen levels and significant coagulopathy. The absence of standardized guidelines complicates decision-making, particularly when anticoagulation is contraindicated due to high hemorrhagic risk. In this case, management required careful use of fibrinogen replacement and supportive therapy, highlighting the need for individualized and multidisciplinary approaches. Given the rarity of such presentations, early recognition, appropriate neuroimaging, and a tailored therapeutic plan are critical. There is an urgent need for larger studies and international collaboration to develop evidence-based protocols that address both bleeding and thrombotic risks in this unique patient population.
